# DeepDist: real-value inter-residue distance prediction with deep residual convolutional network

**DOI:** 10.1186/s12859-021-03960-9

**Published:** 2021-01-25

**Authors:** Tianqi Wu, Zhiye Guo, Jie Hou, Jianlin Cheng

**Affiliations:** 1 Electrical Engineering and Computer Science Department, University of Missouri, Columbia, MO 65211 USA; 2 Department of Computer Science, Saint Louis University, St. Louis, MO 63103 USA

**Keywords:** Protein distance prediction, Contact prediction, Protein structure prediction, Deep learning

## Abstract

**Background:**

Driven by deep learning, inter-residue contact/distance prediction has been significantly improved and substantially enhanced ab initio protein structure prediction. Currently, most of the distance prediction methods classify inter-residue distances into multiple distance intervals instead of directly predicting real-value distances. The output of the former has to be converted into real-value distances to be used in tertiary structure prediction.

**Results:**

To explore the potentials of predicting real-value inter-residue distances, we develop a multi-task deep learning distance predictor (DeepDist) based on new residual convolutional network architectures to simultaneously predict real-value inter-residue distances and classify them into multiple distance intervals. Tested on 43 CASP13 hard domains, DeepDist achieves comparable performance in real-value distance prediction and multi-class distance prediction. The average mean square error (MSE) of DeepDist’s real-value distance prediction is 0.896 Å^2^ when filtering out the predicted distance ≥ 16 Å, which is lower than 1.003 Å^2^ of DeepDist’s multi-class distance prediction. When distance predictions are converted into contact predictions at 8 Å threshold (the standard threshold in the field), the precision of top L/5 and L/2 contact predictions of DeepDist’s multi-class distance prediction is 79.3% and 66.1%, respectively, higher than 78.6% and 64.5% of its real-value distance prediction and the best results in the CASP13 experiment.

**Conclusions:**

DeepDist can predict inter-residue distances well and improve binary contact prediction over the existing state-of-the-art methods. Moreover, the predicted real-value distances can be directly used to reconstruct protein tertiary structures better than multi-class distance predictions due to the lower MSE. Finally, we demonstrate that predicting the real-value distance map and multi-class distance map at the same time performs better than predicting real-value distances alone.

## Background

Recently, the accuracy of protein inter-residue contact prediction has been substantially increased due to the development of residue-residue co-evolution analysis methods effectively detecting the directly correlated mutations of contacted residues in the sequences of a protein family, such as Direct Coupling Analysis (DCA) [1], plmDCA [2], GREMLIN [3], CCMpred [4], and PSICOV [5]. The capability of these methods to extract the correlated mutation information for contact prediction largely depends on the number of effective sequences in multiple sequence alignment (MSA) of a target protein. Due to the advancement in the DNA/RNA sequencing technology [6, 7], many proteins have a lot of sufficiently diverse, homologous sequences that make their contact/distance prediction fairly accurate. However, for targets with a small number of effective homologous sequences (i.e. shallow sequence alignments), the co-evolutionary scores are noisy and not reliable for contact prediction. The problem can be largely addressed by using noisy co-evolutionary scores as input for advanced deep learning techniques that have strong pattern recognition power to predict inter-residue contacts and distances.

After deep learning was introduced for contact prediction in 2012 [8], different deep learning architectures have been designed to integrate traditional sequence features with inter-residue coevolution scores to substantially improve contact/distance prediction [9–12], even for some targets with shallow MSAs.

The improved contact predictions can be converted into inter-residue distance information, which has been successfully used with distance-based modeling methods such as CONFOLD [13], CONFOLD2 [14], and EVFOLD [15] to build accurate tertiary structures for ab initio protein targets [16, 17].

In the most recent CASP13 experiment, several groups (e.g., AlphaFold [18] and RaptorX [19]) applied deep learning techniques to classify inter-residue distances into multiple fine-grained distance intervals (i.e. predict the distance distribution) to further improve ab initio structure prediction substantially. However, the probabilities of a distance belonging to different intervals predicted by the multi-classification approach still need to be converted into a distance value to be used for tertiary structure modeling. There is lack of deep learning regression methods to directly predict the exact real value of inter-residue distances.

In this study, we develop a deep residual convolutional neural network method (DeepDist) to predict both the full-length real-value distance map and the multi-class distance map (i.e. distance distribution map) for a target protein. According to the test on 43 CASP13 hard domains (i.e. both FM and FM/TBM domains; FM: free modeling; TBM: template-based modeling), 37 CASP12 hard (FM) domains, and 268 CAMEO targets, the method can predict inter-residue distance effectively and perform better than existing state-of-the-art methods in terms of the precision of binary contact prediction. We further show that predicting both real-value distance map and multi-class distance map simultaneously is more accurate than only predicting real-value distance map, demonstrating the advantage of DeepDist multi-task learning framework to improve protein distance prediction.

## Results

### Comparing DeepDist with state-of-the-art methods on CASP12 and CASP13 datasets in terms of precision of binary contact predictions

**Fig. 1:**
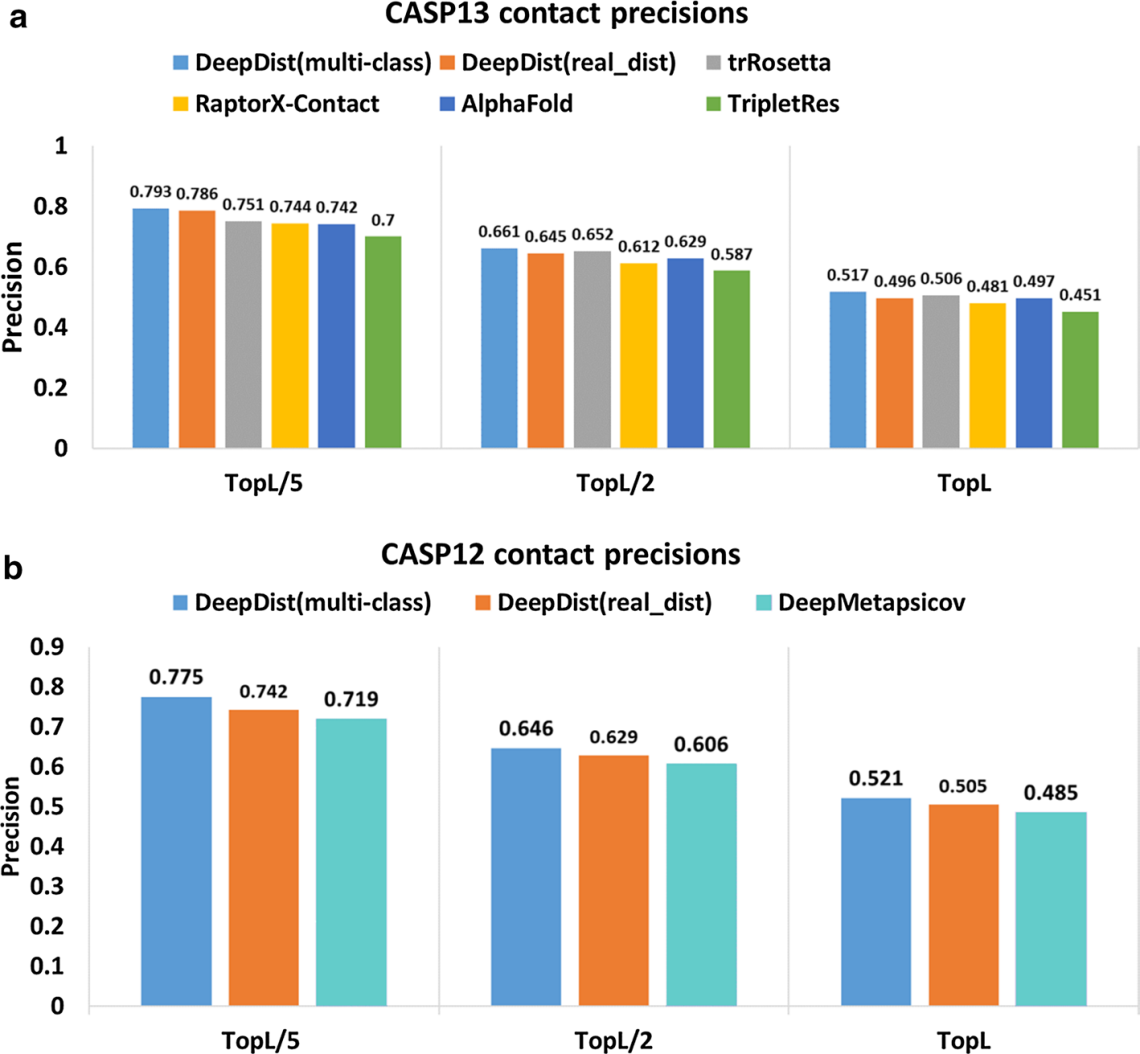
Contact prediction precision of DeepDist and several state-of-art methods on CASP12 and CASP13 test sets. **a** Long-range contact prediction precision of DeepDist, RaptorX-Contact, AlphaFold, TripletRes, and trRosetta on 43 CASP13 FM and FM/TBM domains. “Top L/5”, “Top L/2” and “Top L” stands for the top L/5, L/2 and L predicted contacts, where L is the length of the domain. **b** Long-range contact prediction precision of DeepDist and DeepMetaPSICOV on 37 CASP12 FM domains.

As a multi-task predictor, our distance predictor DeepDist can not only classify each residue pair into distance intervals (multi-classification) but also predict its real-value distance (regression). We convert the predicted distances into contact maps in order to compare DeepDist with existing methods using the most widely used evaluation metrics—the precision of top L/5, L/2, L long-range contact predictions (long range: sequence separation of the residue pair ≥ 24). Figure 1 reports the contact prediction precision of the multi-class distance prediction and the real-value distance prediction of DeepDist and several state-of-the-art methods on two CASP test datasets (43 CASP13 FM and FM/TBM domains and 37 CASP12 FM domains). To compare our distance prediction result on 43 CASP13 test sets strictly, we extract the contact precision results of RaptorX-Contact [19], AlphaFold [18], and TripletRes [12] reported in their paper. For trRosetta [20], we ran it with the same MSAs used with DeepDist to predict distance probability distribution map and converted it into a binary contact map within 8 Å threshold. On the CASP13 dataset (Fig. 1a), the contact precision of DeepDist is higher than the contact precision of three top methods (RaptorX-Contact, AlphaFold, and TripletRes) in CASP13 as well as trRosetta in almost all cases. For instance, the precision of top L/5 long-range predicted contacts for DeepDist(multi-class) and DeepDist(real_dist) is 0.793 and 0.786 on the CASP13 dataset, respectively, higher than 0.751 of trRosetta. The precision of top L/2 long-range predicted contacts for DeepDist(multi-class) is 0.661, which is also similar to trRosetta’s precision—0.652. According to this metric, the multi-class distance prediction (DeepDist(multi-class)) works slightly better than the real-value distance prediction (DeepDist(real_dist)).

We also compare DeepDist with DeepMetaPSICOV [11] on 37 CASP12 FM domains. To rigorously evaluate them, we ran DeepMetaPSICOV with the same sequence-based features (sequence profile from PSI-BLAST [21] and solvent accessibility from PSIPRED [22]) and MSAs used with DeepDist. Both multi-class distance prediction and real-value distance prediction of DeepDist perform consistently better than DeepMetaPSICOV (Fig. 1b).

### Comparison of predicting real-value distance map and multi-class distance map simultaneously with predicting real-value distance map alone

In order to evaluate if predicting real-value distance map and multi-class distance map together improves the performance over predicting real-value distance map only, we conducted two experiments. Experiment 1 trained real-value distance prediction and multi-class distance prediction simultaneously; Experiment 2 trained real-value distance prediction only. To ensure a fair comparison, two experiments used the same input features (PLM) and the same model architecture (PLM_Net mentioned in Method section).

We evaluated the real-value distance prediction performance of the two experiments based on several evaluation metrics—long-range (residue pair separation ≥ 24) contact precision, MSE, and Pearson coefficient. As the evaluation data shown in Table 1, the real-value distance prediction trained simultaneously with multi-class distance prediction in Experiment 1 performed better than the real-value distance prediction trained alone in Experiment 2 according to all the metrics. The results demonstrate that DeepDist’s multi-task learning framework can improve the performance of real-value distance prediction.

**Table 1:** The results of predicting real-value distance map and multi-class distance map at the same time versus predicting real-value distance separately on 43 CASP13 hard domains

	L/5 (Precision)	L/2 (Precision)	L (Precision)	MSE	Pearson coefficient
Experiment 1	0.699	0.580	0.446	1.151	0.979
Experiment 2	0.687	0.558	0.430	1.282	0.978

MSE: average mean square error between predicted distances and true distances; Pearson coefficient: the Pearson’s correlation between predicted distance and true distance

Experiment 1: real-value distance prediction by training real-value distance prediction and multi-class distance prediction simultaneously

Experiment 2: real-value distance prediction by training real-value distance prediction alone. The two experiments used the same input features PLM and the same model architecture PLM_Net

### Comparison of the ensemble model based on four kinds of inputs and a single model based on one input

Table 2 reports the performance of DeepDist (an ensemble of multiple models trained on four kinds of inputs) on the CASP13 dataset. The accuracy of DeepDist’s real-value distance prediction (DeepDist(real-dist)) and multi-class distance prediction (DeepDist(multi-class)) in Table 2 is substantially higher than the accuracy of Experiment 1 in Table 1, a single deep model trained on one kind of feature—PLM. For instance, the precision for top L/5 contact prediction and MSE of DeepDist (real-dist) are 0.786 and 0.896 Å^2^, better than 0.699 and 1.151 Å^2^ of the single model PLM_Net. The same results are observed for other single models trained on COV, PRE, or OTHER features, separately. The results clearly demonstrate that the ensemble approach improves the accuracy of inter-residue distance prediction.

**Table 2:** The performance of DeepDist on 43 CASP13 hard domains

	L/5 (Precision)	L/2 (Precision)	L (Precision)	MSE	Pearson coefficient
DeepDist (real-dist)	0.786	0.645	0.496	0.896	0.981
DeepDist (multi-class)	0.793	0.661	0.517	1.003	0.981

DeepDist(real-dist): real-value distance prediction; DeepDist(multi-class): multi-class distance prediction

### Comparison between real-value distance prediction and multi-class distance distribution prediction in terms of 3D protein structure folding

To test the usefulness of two distance predictions for 3D structure folding, we use the real-value distance map and multi-class distance map predicted by DeepDist with DFOLD [23] to construct the 3D models for the 43 CASP13 hard domains respectively. Table 3 shows the average TM-score of the top 1 model and the best model of the top 5 models of using real-value distances (DeepDist(real-dist)) and of using multi-class distances (DeepDist(multi-class)) on the 43 CASP13 FM and FM/TBM domains. The average TM-scores of top 1 and top 5 models generated from real-value distance predictions are 0.487 and 0.522, which demonstrates the feasibility of applying real-value distance predictions to build protein tertiary structures with moderate model quality.

**Table 3:** TM-scores of models on CASP13 43 FM and FM/TBM domains for four methods

Method	Top 1	Top 5	# of TM-score ≥ 0.5 (Top 1)	# of TM-score ≥ 0.5 (Top 5)
DeepDist (real-dist)	0.487	0.522	21	23
DeepDist (multi-class)	0.463	0.506	21	22
DMPfold	0.438	0.449	16	16
CONFOLD2	0.382	0.466	12	19

**Fig. 2:**
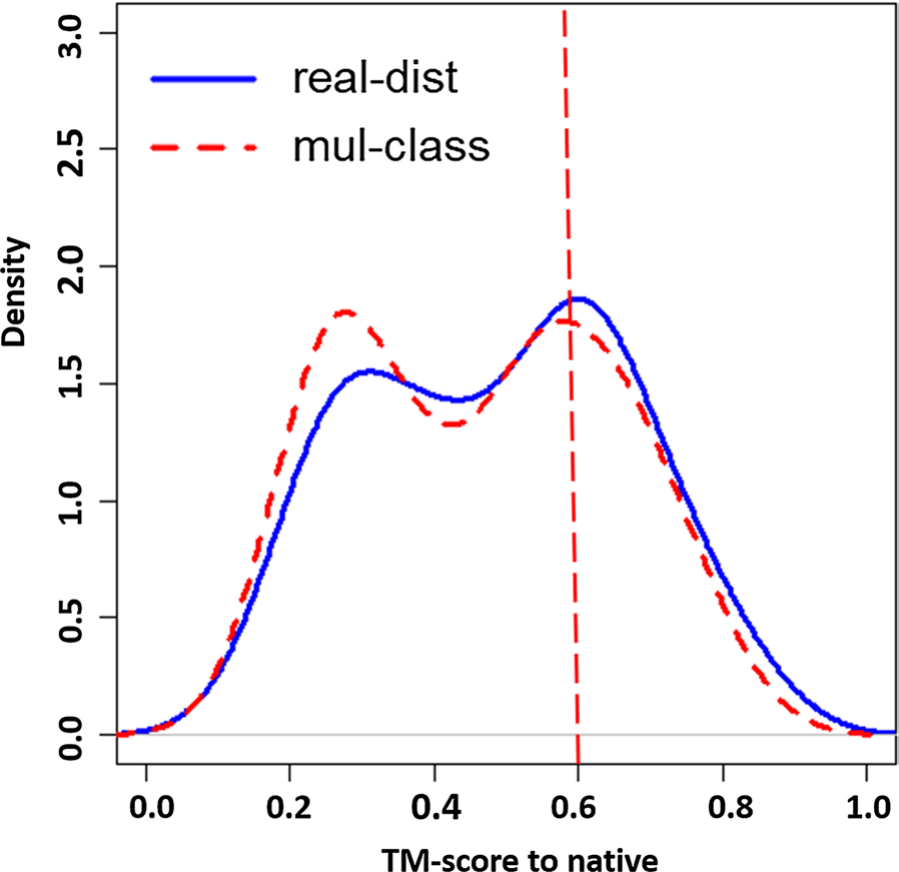
Distribution of TM-scores of the top 1 models of 43 CASP13 FM and FM/TBM domains, built from the real-value distance predictions and the multi-class distance predictions

Figure 2 illustrates the distribution of TM-score of the top1 models of 43 CASP13 domains for DeepDist (real-dist) and DeepDist(multi-class). The distribution of DeepDist (real-dist) shifts toward higher scores (TM-score > 0.6). As shown in Additional file 1: Table S1, the real-value distance prediction has 13 domains with TM-score > 0.6 and the multi-class distance prediction has 12. From the target-by-target comparison, when the models of both methods have TM-score > 0.6, models constructed from the real-value distance prediction tend to have higher scores. This is also consistent with what was observed in Fig. 2, a tendency of the TM-score distribution curve of the real-value distance prediction sitting above the curve of the multi-class distance prediction when TM-score > 0.6. The reduction of MSE of the predicted distances may be one of the factors contributing to the improvement of DeepDist (real-dist) over DeepDist(multi-class) for 3D modeling. The average MSE between the predicted real-value distance map and the true distance map is 0.8964 Å^2^, which is lower than the average MSE (1.0037 Å^2^) between the distance map converted from the predicted multi-class distance map and the true distance map. The way of converting multi-class distance predictions to real-value distance constraints and setting the upper and lower distance bounds for constructing 3D models can be another two factors that affect the final model quality.

**Fig. 3:**
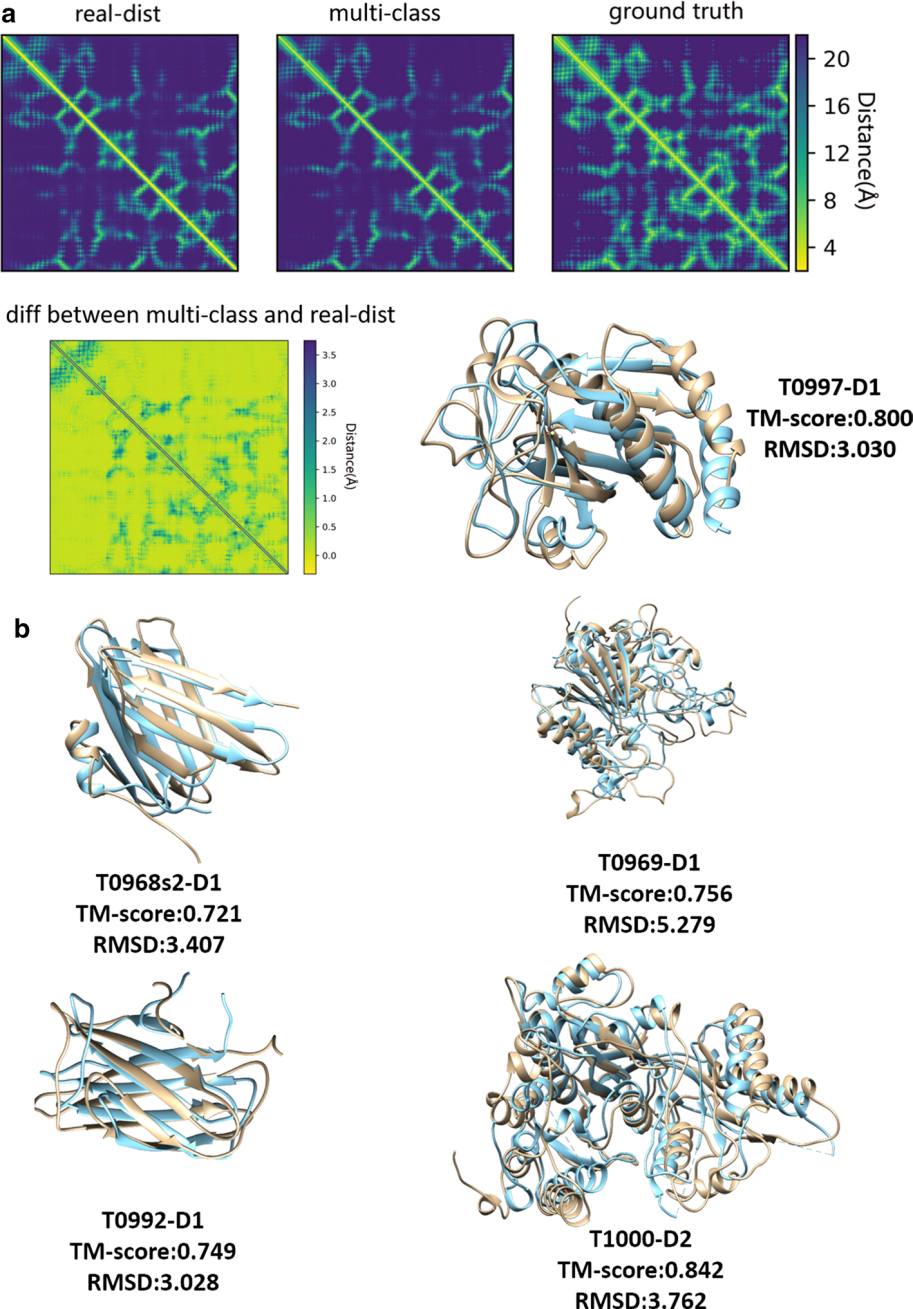
DeepDist predicted distance maps for the target T0997 and the four high-quality tertiary structure models of CASP13 targets (T0968s2-D1, T0969-D1, T0992-D1, T1000-D2) (TM-score ≥ 0.7) generated from DeepDist real-value distance predictions versus their native structures. **a** Two types of distance outputs from DeepDist for the target T0997 are shown as “real-dist” (for real-value distance prediction) and “multi-class” (for multi-class distance prediction). The true distance map of T0997 is marked as “ground truth”. The brightness of each pixel represents the distance of each residue pair of T0997—the brighter the pixel, the shorter the distance. For comparing the two predicted distance maps, the difference of predicted distance maps between “multi-class” and “real-dist” is shown. The brightness of each pixel represents the distance difference between “multi-class” and “real-dist” in each residue pair, i.e., the brighter the pixel, the smaller the distance difference. 3D model comparison is also shown, with the model built from DeepDist real-value distance prediction in brown and the native structure in blue. **b** Model comparison of other four high-quality CASP13 models (TM-score ≥ 0.7) generated from DeepDist real-value distance predictions versus their native structures. Brown: model; Blue: native structure.

On the 43 CASP13 FM and FM/TBM domains, we also compared the models generated from the predicted distance of DeepDist with two popular ab initio distance-based model folding methods: DMPfold [24] and CONFOLD2 [14] (Table 3). For DMPfold, we applied the same sequence-based features and multiple sequence alignment used with DeepDist as input for DMPfold to build 3D models. For CONFOLD2, we converted the predicted distance map to the contact map as its input to build 3D models. As shown in Table 3, Both DeepDist and DMPfold have a much better performance than the contact-based method CONFOLD2, clearly demonstrating that the distance-based 3D modeling is better than contact-based 3D modeling. The average TM-score of DeepDist (real-dist) is 0.487, higher than 0.438 of DMPfold, probably due to more accurate distance prediction made by DeepDist. Considering top 5 models, DeepDist(real_dist) folds 23 out of 43 domains (TM-score > 0.5) correctly, higher than 16 of DMPfold. Figure 3 illustrates the DeepDist distance map for the target T0997 and other four high-quality CASP13 tertiary structure models built from the predicted real-value distances that have the TM-scores ≥ 0.7.

### The relationship between 3D models reconstructed from predicted real-value distances and multiple sequence alignments.

**Fig. 4:**
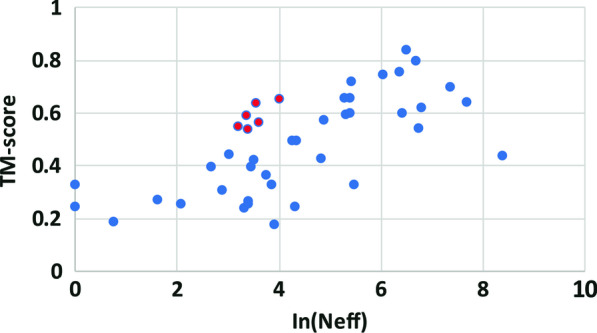
The quality of the top 1 models folded from DeepDist real-value distance predictions versus the logarithm of the number of effective sequences (Neff) on 43 CASP13 FM and FM/TBM domains. The six points in red denote domains with shallow alignments (Neff < 55) but correctly predicted structural folds (TM-score > 0.5)

The main input features used with DeepDist are derived from MSAs. Figure 4 plots the TM-scores of top 1 models of 43 CASP13 domains against the natural logarithm of the number of effective sequences in their MSAs. There is a moderate correlation (Pearson’s correlation = 0.66) between the two. Moreover, 3D models for 6 domains (T0957s2-D1, T0958-D1, T0986s2-D1, T0987-D1, T0989-D1, and T0990-D1) with shallow alignments (the number of effective sequences (Neff) in the alignment < 55) have TM-score > 0.5 (i.e. TM-score 0.568, 0.644, 0.658, 0.555, 0.545 and 0.593, respectively), indicating DeepDist works well on some targets with shallow alignments.

### Evaluation of CAMEO targets

**Fig. 5:**
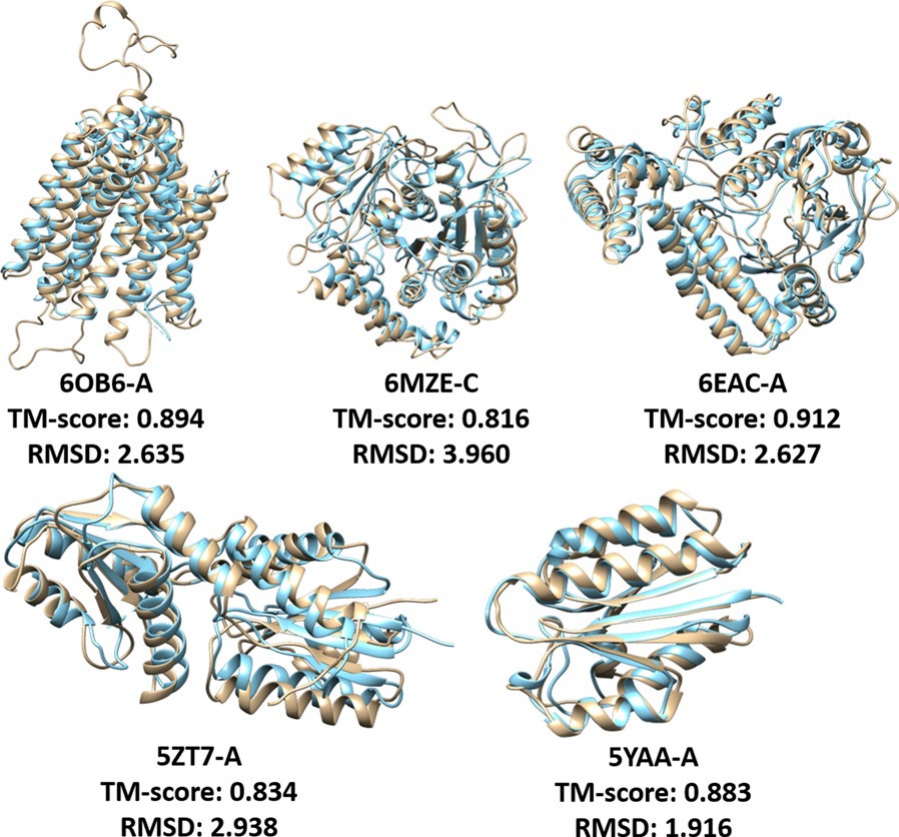
High-quality 3D models for five CAMEO targets constructed from DeepDist predicted real-value distances. The model is shown in brown and the native structure is shown in blue

In order to further evaluate DeepDist on a large dataset, we test DeepDist on 268 CAMEO targets selected from 08/31/2018 to 08/24/2019. The average precision of the top L/5 or L/2 long-range inter-residue contact prediction converted from the real-value distance prediction is 0.691, and 0.598, respectively. 191 out of 268 targets have the long-range top L/5 contact prediction precision ≥ 0.7. Figure 5 shows 5 high-quality models constructed from DeepDist predicted real-value distances. For the 14 targets with the number of effective sequences less than or equal to 50, the average top L/5 and top L/2 long-range contact prediction precision is 0.696 and 0.515, which is reasonable. Using the predicted distance to build 3D structures for the 14 targets, five of them have models with TM-score > 0.5. This further confirms that DeepDist’s predicted distances can fold some proteins with very shallow alignments correctly.

## Discussion

Although there are numerous deep learning methods to conduct distance prediction by classifying distance into multiple intervals, there are few deep learning methods to predict real-value distance via regression. Our results demonstrate that it is worthwhile to explore the potentials of real-value distance prediction, which can be directly used by 3D modeling methods to build protein tertiary structures. Evaluated by the precision of binary contact prediction, the accuracy of predicting real-value distance prediction alone is worse than predicting real-value distances and classifying distances into multiple intervals at the same time in a multi-task learning framework (Table 1). This demonstrates that the strength of DeepDist predicting the two types of distances simultaneously to improve the accuracy of predicting real-value distance. Moreover, the two distance predictions in DeepDist achieve comparable results. The distance multi-classification prediction of DeepDist is slightly better than real-value distance prediction in terms of precision of contact prediction, but it is a little worse in terms of MSE of predicted distance. The p-value (shown in Additional file 1: Tables S2 and S3) calculated from the paired t-test of the corresponding MSE value pairs between DeepDist(real-dist) and DeepDist(multi-class) suggests the significant differences in their mean MSE values. All those results show that the real-value distance prediction can add some value on top of distance multi-classification prediction. Both the strengths and weaknesses of the two distance prediction methods in DeepDist have been demonstrated in this study. Which method should be chosen to use may depend on the specific needs of users and multiple factors such as how to convert multi-classification distances into real-value distances, how to estimate distance errors, and which distances can be used by a 3D modeling tool. Moreover, more experiments are still needed to investigate if and how real-value distance prediction can directly improve the performance of distance multi-classification prediction.

## Conclusion

We develop an inter-residue distance predictor DeepDist based on new deep residual convolutional neural networks to predict both real-value distance map and multi-class distance map simultaneously. We demonstrate that predicting the two at the same time yields higher accuracy in real-value distance prediction than predicting real-value distance alone. The overall performance of DeepDist’s real-value distance prediction and multi-class distance prediction is comparable according to multiple evaluation metrics. Both kinds of distance predictions of DeepDist are more accurate than several state-of-the-art methods on the CASP13 hard targets. Moreover, DeepDist can work well on some targets with shallow multiple sequence alignments. And the real-value distance predictions can be used to reconstruct 3D protein structures better than predicted multi-class distance predictions, showing that predicting real-value inter-residue distances can add the value on top of existing distance prediction approaches.

## Methods

### Overview

**Fig. 6:**
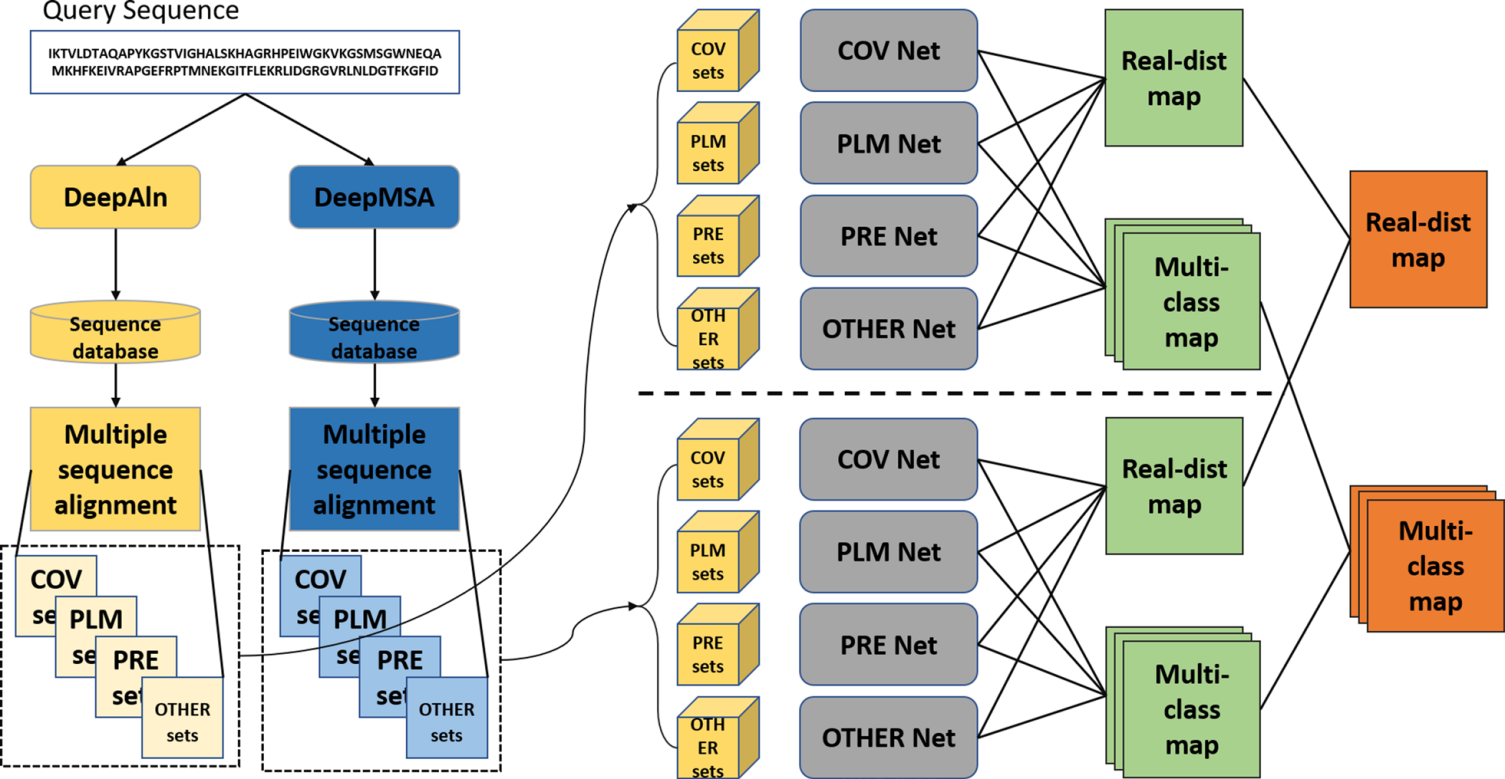
The overall workflow of DeepDist for both real-value distance map prediction and multi-class distance map prediction. Given a sequence, DeepAln and DeepMSA are called to search it against sequence databases to generate two kinds of multiple sequence alignments (MSAs), which are used to generate four sets of features (COV, PLM, PRE, OTHER), respectively. The four sets of features are used by four deep networks (COV Net, PLM Net, PRE Net, and OTHER Net) to predict both real-value distance (real-dist) map and multi-class distance (multi-class) map, respectively. The real-value distance maps (or multi-class distance maps) of the individual networks are averaged to produce the final real-value distance map (or multi-class distance map)

The overall workflow of DeepDist is shown in Fig. 6. We use four sets of 2D co-evolutionary and sequence-based features to train four deep residual convolutional neural network architectures respectively to predict the Euclidean distance between residues in a protein target. Three of four feature sets are mostly coevolution-based features, i.e. covariance matrix (COV) [25], precision matrix (PRE) [26], and pseudolikelihood maximization matrix (PLM) [4]) calculated from multiple sequence alignments. Considering that coevolution-based features sometimes cannot provide sufficient information, particularly when targets have shallow alignments, the fourth set of sequence-based features (OTHER), such as the sequence profile generated by PSI-BLAST [21], and solvent accessibility from PSIPRED [22] are used. The output of DeepDist is a real-value L × L distance map and a multi-class distance map (L: the length of the target protein). The two types of distance maps are generated by two prediction branches. For each branch, the final output is produced by the ensemble of four deep network models (COV_Net, PLM_Net, PRE_Net, and OTHER_Net) named after their input feature sets (COV, PLM, PRE, and OTHER). For the prediction of the multi-class distance map, we discretize the inter-residue distances into 25 bins: 1 bin for distance < 4.5 Å, 23 bins from 4.5 to 16 Å at interval size of 0.5 Å and a final bin for all distances ≥ 16 Å. For the real-value distance map, we simply use the true distance map of the native structure as targets to train deep learning models without discretization. Because large distances are not useful and not predictable, we only predict inter-residue distances less than 16 Å by filtering out true distances ≥ 16 Å.

### Datasets

We select targets from the training list used in DMPfold [24] and extract their true structures from the Protein Data Bank (PDB) to create a training dataset. After filtering out the redundancy with the validation dataset and test datasets according to 25% sequence identity threshold, 6463 targets are left in the training dataset. The validation set contains 144 targets used to validate DNCON2 [10]. The three blind test datasets are 37 CASP12 FM domains, 43 CASP13 FM and FM/TBM domains, and 268 CAMEO targets collected from 08/31/2018 to 08/24/2019.

### Input feature generation

The sequence databases used to search for homologous sequences for feature generation include Uniclust30 (2017-10) [27], Uniref90 (2018-04), Metaclust50 (2018-01) [28], a customized database that combines Uniref100 (2018-04) and metagenomics sequence databases (2018-04), and NR90 database (2016). All the sequence databases were constructed before the CASP13 experiment.

Co-evolutionary features (i.e. COV, PRE, and PLM) are the main input features for DeepDist, where COV is the covariance matrix calculated from marginal and pair frequencies of each amino acid pair [25], PRE [26] is the inverse covariance matrix, and PLM is the inverse Potts model coupling matrix optimized by pseudolikelihoods [4]. All the three coevolutionary features are generated from multiple sequence alignment (MSA). Two methods, DeepMSA [29] and our in-house DeepAln, are used to generate MSA for a target. The outputs of both MSA generation methods are the combination of the iterative homologous sequence search of HHblits [30] and Jackhmmer [31] on several sequence databases. The two methods differ in sequence databases used and the strategy of combining the output of HHblits and Jackhmmer searches. DeepMSA trims the sequence hits from Jackhmmer and performs sequence clustering, which shortens the time for constructing the HHblits database for the next round of search. To leverage its fast speed, we apply DeepMSA to search against a large customized sequence database that is composed of UniRef100 and metagenomic sequences. In contrast, DeepAln directly uses the full-length Jackhmmer hits for building HHblits customized databases and is slower. It is applied to the Metaclust sequences database. The detailed comparison of two MSA generation methods is reported in the Additional file 1: Table S4. In addition to three kinds of co-evolutionary features, 2D features such as the coevolutionary contact scores generated by CCMpred, Shannon entropy sum, mean contact potential, normalized mutual information, and mutual information are also generated. Moreover, some other features used in DNCON2 including sequence profile, solvent accessibility, joint entropy, and Pearson correlation are also produced, which are collectively called OTHER feature.

The features above are generated for the MSAs of both DeepMSA and DeepAln. Each of them is used to train a deep model to predict both real-value distance map and multi-class distance map, resulting in 8 predicted real-value distance maps and 8 multi-class distance maps (Fig. 6).

### Deep network architectures for distance prediction

**Fig. 7:**
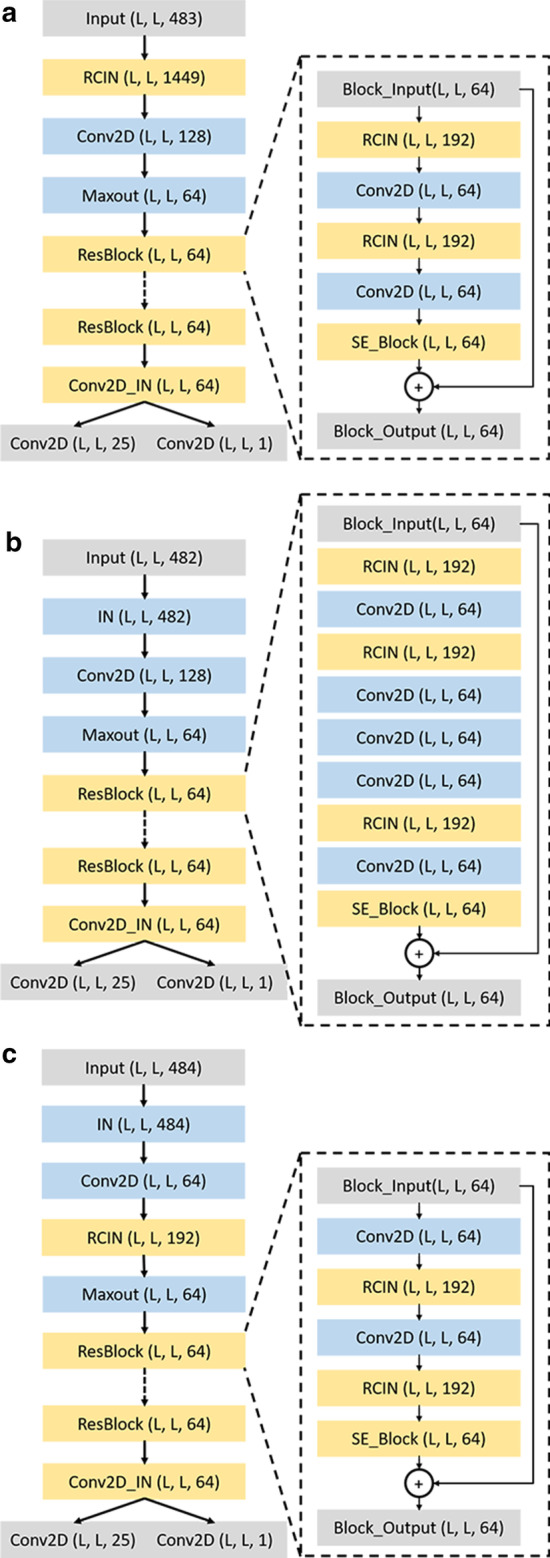
Deep network architectures for four deep residual network models. **a** COV_Net; **b** PLM_Net; **c** PRE_Net/OTHER_Net. RCIN: normalization layer; SE_block: squeeze-and-excitation block

We started training the first network (COV_Net) with a simple feature set which consists of the covariance matrix described above, along with sequence profile (PSSM), contact scores (CCMpred), and Pearson correlation. Inspired by COV_Net, two networks—PLM_Net and PRE_Net that use two related coevolutionary matrices PLM and PRE generated from multiple sequence alignment were then added to use the coevolutionary relationship between amino acid pairs more effectively. Since all three networks highly depend on the quality of MSA, the fourth network OTHER_Net was constructed by adding only non-coevolutionary sequence-based features as input in case the MSA is shallow. To make sure every network works well, we tweaked the model architecture for each feature set. In total, there are four different networks in DeepDist, which are called COV_Net, PLM_Net, PRE_Net, and OTHER_Net (Fig. 7), respectively. PRE_Net and OTHER_Net share almost the same architecture with some minor differences. The detailed comparison of four networks is shown in Additional file 1: Table S5.

COV_Net (Fig. 7a) uses the COV matrix along with sequence profile (PSSM), contact scores (CCMpred), and Pearson correlation as input. It starts with a normalization block called RCIN that contains instance normalization (IN) [32], row normalization (RN), column normalization (CN) [33] and a ReLU [34] activation function, followed by one convolutional layer with 128 kernels of size 1 × 1 and one Maxout [35] layer to reduce the input channel from 483 to 64. The output of Maxout is then fed into 16 residual blocks. Each residual block is composed of two RCIN normalization blocks, two convolutional layers that consist of 64 kernels of size 3 × 3, and one squeeze-and-excitation block (SE_block) [36]. The output feature maps from the block, together with the input of the block are added together as input for a ReLU activation function to generate the output of the residual block. The last residual block is followed by one convolutional instance normalization layer. The output of the layer is converted into two output maps simultaneously. One real-value distance map is obtained by a ReLU function through a convolution kernel of size 1 × 1, and one multi-class distance map with 25 output channels is obtained by a softmax function.

PLM_Net (Fig. 7b) uses as input the PLM matrix concatenated with the sequence profile (PSSM) and Pearson correlation. The input is first fed into an instance normalization layer, followed by one convolutional layer and one Maxout layer. The output of Maxout is then fed into 20 residual blocks. Each residual block contains three RCIN blocks, four convolutional layers with 64 kernels of size 3 × 3, one SE_block, and one dropout layer [37] with a dropout rate of 0.2. The residual block is similar to the bottleneck residual block, except that the middle convolutional layer of kernel size 3 × 3 is replaced with three convolutional layers of kernel size 3 × 3, 7 × 1, 1 × 7, separately. The last residual block is followed by the same layers as in COV_Net to predict a real-value distance map and a multi-class distance map.

PRE_Net (Fig. 7c) uses as input the PRE matrix as well as entropy scores (joint entropy, Shannon entropy) and sequence profile (PSSM). An instance normalization layer is first applied to the input. Unlike COV_Net and PLM_Net, one convolutional layer with 64 kernels of size 1 × 1 and an RCIN block are applied after the instance normalization layer for dimensionality reduction. The output of the RCIN block is then fed through 16 residual blocks. Each residual block is made of two stacked sub-blocks (each containing one convolutional layer with 64 kernels of size 3 × 3, an RCIN block, a dropout layer with a dropout rate of 0.2, a SE_block, and the shortcut connection). The final output layers after the residual blocks are the same as in COV_Net.

OTHER_Net uses OTHER features as input. Its architecture is basically the same as PRE_Net, except that it has 22 residual blocks and there is no dropout layer in each residual block.

The final output of DeepDist is an average real-value distance map and an average multi-class distance map calculated from the output of the four individual network models, i.e. the output of the ensemble of the individual networks.

### Training

The dimension of the input of COV_Net, PLM_Net, and PRE_Net is L × L × 483, L × L × 482, and L × L × 484 respectively, which is very large and consumes a lot of memory. Therefore, we use data generators from Keras to load large feature data batch by batch. The batch size is set as 1. A normal initializer [38] is used to initialize the network. For epochs ≤ 30, Adam optimizer [39] is performed with an initial learning rate of 0.001. For epochs > 30, stochastic gradient descent (SGD) with momentum [40] is used instead, with the initial learning rate of 0.01 and the momentum of 0.9. The real-value distance prediction and multi-class distance classification are trained in two parallel branches. The mean squared error (MSE) and cross-entropy are used as their loss function, respectively. At each epoch, the precision of top L/2 long-range contact predictions derived from the average of the two contact maps converted from the real-value distance map and the multi-class distance map on the validation dataset is calculated. The inter-residue real-value distance map is converted to the contact map by inversing the predicted distance to obtain a relative contact probability (i.e. 1/dij: relative contact probability score; dij: predicted distance between residues i and j). The multi-class distance map is converted to the binary contact map by summing up the predicted probabilities of all the distance intervals ≤ 8 Å as contact probabilities.

### Ab initio protein folding by predicted distances

We use distances predicted by DeepDist with our in-house tool—DFOLD [23] built on top of CNS [41], a software package that implements distance geometry algorithm for NMR based structure determination, to convert the distance restraints into 3D structure models. For the predicted real-value distance map, we select the predicted distances ≤ 15 Å and with sequence separation ≥ 3 to generate the distance restraints between Cb-Cb atoms of residue pairs. 0.1 Å is added to or subtracted from the predicted distances to set the upper and lower distance bounds. For the predicted multi-class distance map, we first convert the distance probability distribution matrix to a real-value distance map by setting each distance as the probability-weighted mean distance of all intervals for a residue pair and using the standard deviation to calculate the upper and lower distance bounds. Given a final real-value distance map, we prepare five different subsets of input distance restraints by filtering out distances ≥ x respectively, where x = 11 Å, 12 Å, 13 Å, 14 Å, and 15 Å. For each subset of distance restraints, we run DFOLD for 3 iterations. For each iteration, we generate 50 models and select the top five models ranked by the CNS energy score, the sum of all violations of all distance restraints used to generate a model. The top selected models generated from five subsets are further ranked by SBROD [42]. The final top one model is the one with the highest SBROD score. PSIPRED is used to predict the secondary structure to generate hydrogen bonds and torsion angle constraints for DFOLD to use.

## Supplementary information

**Additional file 1.** Supplemental results and data.

## Data Availability

The datasets used in this study and the source code of DeepDist are available at https://github.com/multicom-toolbox/deepdist.

## Abbreviations

MSE: The average mean square error
DCA: Direct coupling analysis
MSA: Multiple sequence alignment
FM: Free modeling
TBM: Template-based modeling
