# Correction to: DeepDist: real‑value inter‑residue distance prediction with deep residual convolutional network

**DOI:** 10.1186/s12859-021-04269-3

**Published:** 2021-06-29

**Authors:** Tianqi Wu, Zhiye Guo, Jie Hou, Jianlin Cheng

**Affiliations:** 1 Electrical Engineering and Computer Science Department, University of Missouri, Columbia, MO 65211 USA; 2 Department of Computer Science, Saint Louis University, St. Louis, MO 63103 USA

## Correction to: BMC Bioinformatics (2021) 22:30 10.1186/s12859-021-03960-9

Following the publication of the original article [1], the authors identified that the grant of the funding note is incorrect. The correct funding note is given below.

Funding note

Research reported in this publication was supported in part by two NSF Grants (DBI 1759934 and IIS1763246), a DOE grant(DE-SC0021303) and an NIH Grant (R01GM093123) to JC. The funding agencies did not play a role in this research.

The original article [1] has been corrected.

## References

[1] Wu et al. BMC Bioinformatics (2021) DeepDist: real‑value inter‑residue distance prediction with deep residual convolutional network (2021) 22:30 DOI: 10.1186/s12859‑021‑03960‑9 10.1186/s12859-021-03960-9 PMC7831258 33494711

