# Supplementary material for: DeepDist: real-value inter-residue distance prediction with deep residual convolutional network
Source: BMC Bioinformatics. 2021 Jan 25;22:30. doi: 10.1186/s12859-021-03960-9 (PMC7831258; doi:10.1186/s12859-021-03960-9)
Supplement: Supplementary file 1 — Additional file 1. Supplemental results and data. [file 12859_2021_3960_MOESM1_ESM.docx]

**Table S1 TM-scores of the top 1 models of 43 CASP13 FM and FM/TBM domains, built from DeepDist real-value distance prediction and DeepDist multi-class distance predictions.**

|  | DeepDist(real-dist) | DeepDist(multi-class) |
| --- | --- | --- |
| T1000-D2 | 0.842 | 0.797 |
| T0997-D1 | 0.800 | 0.755 |
| T0969-D1 | 0.756 | 0.533 |
| T0992-D1 | 0.749 | 0.748 |
| T0968s2-D1 | 0.721 | 0.715 |
| T1005-D1 | 0.702 | 0.673 |
| T0986s1-D1 | 0.660 | 0.641 |
| T0986s2-D1 | 0.658 | 0.667 |
| T0953s2-D2 | 0.656 | 0.674 |
| T0958-D1 | 0.644 | 0.649 |
| T0949-D1 | 0.642 | 0.663 |
| T0978-D1 | 0.622 | 0.525 |
| T1019s1-D1 | 0.602 | 0.540 |
| T1022s1-D1 | 0.600 | 0.526 |
| T1015s1-D1 | 0.593 | 0.566 |
| T0990-D1 | 0.593 | 0.236 |
| T0968s1-D1 | 0.573 | 0.544 |
| T0957s2-D1 | 0.568 | 0.575 |
| T0987-D1 | 0.555 | 0.547 |
| T0989-D1 | 0.545 | 0.268 |
| T1021s3-D1 | 0.544 | 0.649 |
| T1010-D1 | 0.496 | 0.416 |
| T1017s2-D1 | 0.495 | 0.575 |
| T0963-D2 | 0.447 | 0.328 |
| T0975-D1 | 0.439 | 0.486 |
| T0950-D1 | 0.431 | 0.575 |
| T0970-D1 | 0.422 | 0.460 |
| T0957s1-D1 | 0.396 | 0.407 |
| T1001-D1 | 0.395 | 0.395 |
| T0989-D2 | 0.367 | 0.259 |
| T0980s1-D1 | 0.332 | 0.335 |
| T1008-D1 | 0.329 | 0.326 |
| T0953s2-D3 | 0.329 | 0.330 |
| T0960-D2 | 0.311 | 0.383 |
| T0981-D3 | 0.271 | 0.245 |
| T0990-D3 | 0.265 | 0.256 |
| T0998-D1 | 0.259 | 0.270 |
| T0990-D2 | 0.257 | 0.254 |
| T0991-D1 | 0.246 | 0.220 |
| T1021s3-D2 | 0.245 | 0.236 |
| T0987-D2 | 0.241 | 0.266 |
| T0981-D2 | 0.188 | 0.185 |
| T0953s2-D1 | 0.177 | 0.220 |
| AVERAGE | 0.487 | 0.463 |

**Table S2 MSE values of CASP13 FM targets for two methods, DeepDist(real-dist) and DeepDist(multi-class),** where Δd is the difference between MSE values from DeepDist(real-dist) and DeepDist(multi-class).

| target | DeepDist(real-dist) | DeepDist(multi-class) | Δd = MSE(real-dist) - MSE(multi-class) |
| --- | --- | --- | --- |
| T1019s1 | 1.525 | 1.602 | -0.077 |
| T1015s1 | 0.424 | 0.434 | -0.011 |
| T1022s1 | 0.325 | 0.346 | -0.021 |
| T0992 | 0.639 | 0.675 | -0.036 |
| T0970 | 1.241 | 1.334 | -0.093 |
| T0986s1 | 0.935 | 1.020 | -0.086 |
| T0953s2 | 1.480 | 1.553 | -0.073 |
| T1021s3 | 0.659 | 0.698 | -0.040 |
| T0989 | 0.727 | 0.928 | -0.201 |
| T0997 | 0.817 | 0.952 | -0.135 |
| T1005 | 0.524 | 0.587 | -0.063 |
| T0949 | 0.529 | 0.657 | -0.128 |
| T0978 | 0.526 | 0.628 | -0.102 |
| T1017s2 | 1.488 | 1.605 | -0.117 |
| T0969 | 0.270 | 0.339 | -0.070 |
| T0980s1 | 1.450 | 1.586 | -0.136 |
| T1000 | 0.217 | 0.265 | -0.049 |
| T0958 | 0.596 | 0.660 | -0.064 |
| T0975 | 0.599 | 0.668 | -0.070 |
| T0990 | 0.469 | 0.630 | -0.160 |
| T0968s1 | 0.913 | 1.018 | -0.105 |
| T0957s1 | 1.141 | 1.299 | -0.159 |
| T0957s2 | 0.976 | 1.076 | -0.100 |
| T1008 | 2.798 | 2.947 | -0.149 |
| T1001 | 1.832 | 2.039 | -0.207 |
| T0987 | 1.005 | 1.136 | -0.131 |
| T0981 | 0.287 | 0.447 | -0.159 |
| T0968s2 | 0.810 | 0.861 | -0.050 |
| T0950 | 0.419 | 0.462 | -0.043 |
| T0986s2 | 1.074 | 1.196 | -0.123 |
| T0998 | 0.747 | 0.863 | -0.116 |
| T0991 | 1.725 | 1.890 | -0.165 |
| T1010 | 1.588 | 1.917 | -0.330 |
| T0960 | 0.312 | 0.410 | -0.098 |
| T0963 | 0.312 | 0.404 | -0.092 |

|  | DeepDist  (real-dist) | DeepDist  (multi-class) | Δd | H0: Δd = 0 |
| --- | --- | --- | --- | --- |
| Mean | 0.896 | 1.004 | -0.107 |  |
| P(T<=t) two-tail | 6.727E-12 |  |  |  |

On CASP13, we applied the paired t-test on two sets of MSE values from DeepDist(real-dist) and DeepDist(multi-class). In this way, we try to focus only on pairwise comparison for each target and remove the uncontrollable factor, structural differences between different targets. Our null hypothesis is that the difference of MSE values from two methods in means is zero, which is no obvious difference in MSE values between the two methods. Given the significance level of 0.05, we have p-value of 6.727E-12 < 0.05 that suggests rejecting the null hypothesis of no difference and indicates the difference exists between MSE values of two methods. From the average MSE differences -0.107, we can further infer that the MSE value in means from DeepDist(real-dist) is less than the one from DeepDist(multi-class). The same analysis is applied to 31 CASP12 FM targets shown in Table S2.

**Table S3 MSE values of CASP12 FM targets for two methods, DeepDist(real-dist) and DeepDist(multi-class),** where Δd is the difference between MSE values from DeepDist(real-dist) and DeepDist(multi-class).

| target | DeepDist(real-dist) | DeepDist(multi-class) | Δd = MSE(real-dist) - MSE(multi-class) |
| --- | --- | --- | --- |
| T0859 | 1.235 | 1.483 | -0.248 |
| T0862 | 0.491 | 0.539 | -0.048 |
| T0863 | 0.231 | 0.333 | -0.102 |
| T0864 | 0.751 | 0.792 | -0.041 |
| T0866 | 0.154 | 0.163 | -0.009 |
| T0868 | 0.599 | 0.669 | -0.070 |
| T0869 | 0.477 | 0.542 | -0.065 |
| T0870 | 0.712 | 0.766 | -0.054 |
| T0878 | 0.776 | 0.824 | -0.047 |
| T0880 | 1.890 | 2.126 | -0.236 |
| T0884 | 0.918 | 1.005 | -0.086 |
| T0886 | 0.228 | 0.239 | -0.011 |
| T0890 | 0.912 | 0.985 | -0.073 |
| T0892 | 0.683 | 0.718 | -0.035 |
| T0894 | 0.389 | 0.416 | -0.027 |
| T0896 | 1.006 | 1.218 | -0.212 |
| T0897 | 1.752 | 1.894 | -0.142 |
| T0898 | 0.965 | 1.082 | -0.116 |
| T0899 | 0.395 | 0.434 | -0.039 |
| T0900 | 1.328 | 1.479 | -0.151 |
| T0901 | 0.605 | 0.638 | -0.033 |
| T0904 | 0.536 | 0.591 | -0.055 |
| T0905 | 0.653 | 0.693 | -0.040 |
| T0912 | 0.311 | 0.413 | -0.101 |
| T0914 | 1.077 | 1.152 | -0.075 |
| T0915 | 0.981 | 1.082 | -0.101 |
| T0918 | 0.167 | 0.205 | -0.038 |
| T0941 | 0.899 | 1.081 | -0.182 |
| T0943 | 0.190 | 0.236 | -0.046 |
| T0945 | 0.380 | 0.411 | -0.032 |
| T0946 | 0.796 | 0.829 | -0.033 |

|  | DeepDist  (real-dist) | DeepDist  (multi-class) | Δd | H0: Δd = 0 |
| --- | --- | --- | --- | --- |
| Mean | 0.725 | 0.808 | -0.082 |  |
| P(T<=t) two-tail | 6.641E-08 |  |  |  |

**Table S4 Comparison of two alignment generation methods DeepAln and DeepMSA on CASP13 FM and FM-TBM domains.** Two methods use the same sequence databases (Uniref90_04_2018, Uniclust30_10_2017, Metaclust50_01_2018). Top L/2 long-range contact precision is shown for those domains. N and Neff values are calculated on the MSA for the full-length target sequence, which means different domains of the same target have the same N and Neff.

| Domain | Precision  (DeepAln)  (%) | Precision  (DeepMSA) (%) | N  (DeepAln) | N  (DeepMSA) | Neff  (DeepAln) | Neff  (DeepMSA) |
| --- | --- | --- | --- | --- | --- | --- |
| T0949-D1 | 100 | 98.46 | 19739 | 5973 | 1325 | 942 |
| T0950-D1 | 80.12 | 76.02 | 329 | 341 | 120 | 118 |
| T0953s2-D1 | 45.45 | 40.91 | 499 | 337 | 195 | 121 |
| T0957s2-D1 | 44.87 | 41.03 | 86 | 76 | 34 | 31 |
| T0958-D1 | 76.92 | 41.03 | 93 | 63 | 29 | 15 |
| T0960-D2 | 14.29 | 11.9 | 124 | 147 | 54 | 59 |
| T0963-D2 | 12.2 | 9.76 | 107 | 134 | 42 | 57 |
| T0968s1-D1 | 45.76 | 40.68 | 262 | 223 | 125 | 90 |
| T0968s2-D1 | 29.31 | 48.28 | 492 | 428 | 224 | 198 |
| T0969-D1 | 89.27 | 90.4 | 8068 | 8156 | 247 | 177 |
| T0970-D1 | 65.12 | 76.74 | 692 | 639 | 33 | 32 |
| T0975-D1 | 65.25 | 63.83 | 10863 | 4948 | 3411 | 905 |
| T0978-D1 | 70.53 | 58.94 | 12623 | 6006 | 855 | 319 |
| T0980s1-D1 | 61.54 | 59.62 | 223 | 197 | 45 | 41 |
| T0981-D3 | 52.94 | 50.98 | 10 | 10 | 4 | 4 |
| T0986s1-D1 | 60.87 | 58.7 | 789 | 771 | 186 | 194 |
| T0986s2-D1 | 75.64 | 67.95 | 102 | 98 | 54 | 51 |
| T0987-D1 | 93.48 | 92.39 | 117 | 134 | 25 | 28 |
| T0987-D2 | 78.57 | 68.37 | 117 | 134 | 25 | 28 |
| T0989-D1 | 53.73 | 53.73 | 53 | 29 | 36 | 24 |
| T0989-D2 | 26.79 | 1.79 | 53 | 29 | 36 | 24 |
| T0990-D1 | 36.84 | 34.21 | 77 | 46 | 30 | 29 |
| T0990-D3 | 36.45 | 33.64 | 77 | 46 | 30 | 29 |
| T0992-D1 | 90.74 | 96.3 | 1475 | 1564 | 304 | 341 |
| T0997-D1 | 89.25 | 90.32 | 6958 | 4624 | 299 | 151 |
| T0998-D1 | 45.78 | 46.99 | 8 | 7 | 8 | 7 |
| T1000-D2 | 93.48 | 90.76 | 5594 | 3354 | 617 | 645 |
| T1001-D1 | 38.57 | 40 | 18 | 18 | 14 | 14 |
| T1005-D1 | 82.82 | 90.8 | 17100 | 14774 | 1102 | 1595 |
| T1008-D1 | 64.1 | 64.1 | 1 | 1 | 1 | 1 |
| T1010-D1 | 67.62 | 69.52 | 119 | 87 | 70 | 52 |
| T1015s1-D1 | 86.36 | 90.91 | 429 | 398 | 203 | 178 |
| T1017s2-D1 | 38.1 | 36.51 | 349 | 374 | 74 | 71 |
| T1019s1-D1 | 27.59 | 31.03 | 768 | 1960 | 221 | 411 |
| T1021s3-D1 | 89.16 | 87.95 | 2723 | 2903 | 293 | 243 |
| T1021s3-D2 | 16.33 | 16.33 | 2723 | 2903 | 293 | 243 |
| T1022s1-D1 | 87.18 | 80.77 | 1561 | 3869 | 559 | 1021 |
| AVERAGE | 60.35 | 58.15 | 2578.95 | 1778.41 | 303.32 | 229.43 |

**Table S5 Mean long-range contact precision of DeepDist real-value distance prediction(a) and multi-class distance prediction(b) achieved by DeepDist four networks on 43 CASP13 FM and FM/TBM domains**.

(a) Mean long-range contact precision of DeepDist(real-dist) 一DeepDist real-value distance prediction achieved by DeepDist four networks on 43 CASP13 FM and FM/TBM domains.

| DeepDist  (real-dist) | Mean precision | | |
| --- | --- | --- | --- |
|  | Top-L/5 | Top-L/2 | Top-L |
| COV_Net | 0.731 | 0.608 | 0.472 |
| PLM_Net | 0.752 | 0.637 | 0.491 |
| PRE_Net | 0.734 | 0.619 | 0.473 |
| OTHER_Net | 0.709 | 0.568 | 0.447 |

(b) Mean long-range contact precision of DeepDist(multi-class) 一DeepDist multi-class distance prediction achieved by DeepDist four networks on 43 CASP13 FM and FM/TBM domains.

| DeepDist  (multi-class) | Mean precision | | |
| --- | --- | --- | --- |
|  | Top-L/5 | Top-L/2 | Top-L |
| COV_Net | 0.731 | 0.611 | 0.473 |
| PLM_Net | 0.739 | 0.636 | 0.494 |
| PRE_Net | 0.721 | 0.611 | 0.477 |
| OTHER_Net | 0.694 | 0.565 | 0.449 |
